# Metastatic Endocrine Mucin-Producing Sweat Gland Carcinoma to the Lung: A Case Report

**DOI:** 10.7759/cureus.49711

**Published:** 2023-11-30

**Authors:** Jeffrey E Fournier, Crispin Russell, Mohammad Hossain

**Affiliations:** 1 Department of Pathology and Molecular Medicine, McMaster University, Hamilton, CAN; 2 Division of Thoracic Surgery, Saint John Regional Hospital, Saint John, CAN; 3 Department of Pathology, Saint John Regional Hospital, Saint John, CAN

**Keywords:** rare, metastasis, mucinous, neuroendocrine, empsgc

## Abstract

Endocrine mucin-producing sweat gland carcinoma (EMPSGC) is a rare, low-grade neuroendocrine neoplasm previously believed to be indolent in nature. There have only previously been six reported cases of metastases and none of thoracic structures. This case shows a metastatic EMPSGC in a 72-year-old male with a complex oncologic history and is the first reported case of metastases to the lung. As increased recognition of this entity continues to grow, it is important to consider it as part of the differential in mucinous and/or neuroendocrine neoplasms for appropriate management. This case adds to the oncologic literature by demonstrating a rare cutaneous neoplasm and emphasizing its metastatic potential.

## Introduction

Endocrine mucin-producing sweat gland carcinoma (EMPSGC) is a rare low-grade cutaneous neuroendocrine neoplasm believed to be of sweat gland origin, the cutaneous analogue to solid papillary adenocarcinoma of the breast, and a potential precursor to mucinous adenocarcinoma of the skin [1-3]. This entity was first described in 1997 [4], and to date there have only been just over 110 cases reported, with a predilection for females in their sixth to seventh decades of life and arising mainly in the periorbital skin [2,3,5]. The current suggested treatment is surgical excision alone [6]. Prior to 2020, this entity was believed to be non-metastasizing, and since then there have only been six reported cases of metastatic disease [3,7-11], primarily to the parotid gland, with one also showing metastases to a rib and one with more systemic involvement. All cases have occurred after a prolonged clinical interval. This case report demonstrates the first known metastases to the lung of a rare cutaneous tumour previously believed to be more indolent in nature.

## Case presentation

A 72-year-old male presented to the emergency department in July 2023 with right flank pain. Computed tomography (CT) imaging was performed, demonstrating a ruptured renal cyst. An incidental finding of a multi-lobulated solid left lower lobe pulmonary nodule measuring 1.8 cm × 1.4 cm × 1.1 cm was identified and suggests a pulmonary neoplasm. The nodule had increased in size on review of a prior imaging study performed in 2021. His physical examination was unremarkable, including no palpable lymphadenopathy, and he was otherwise well apart from the flank pain.

The patient has a complex oncologic history, which includes a remote history of a thymic tumour treated with radiation in 1951, follicular thyroid carcinoma treated with a hemithyroidectomy and radioactive iodine therapy in 1995, prostate acinar adenocarcinoma Gleason 6 treated with prostatectomy in 2003, followed by recurrence treated with radiation in 2009, and an intraductal papillary carcinoma of the left breast treated with mastectomy and radiation in 2011. Most recently, he was diagnosed with a primary cutaneous mucinous carcinoma of the right eyelid in 2015 and treated with surgery and radiation. With the aid of increased recognition of this extremely rare entity and on review of the excised specimen submitted in toto in 2015 in comparison to the current biopsy, the lesion from 2015 was reclassified as an EMPSGC. He has been followed annually and has had no issues until now.

**Figure 1:**
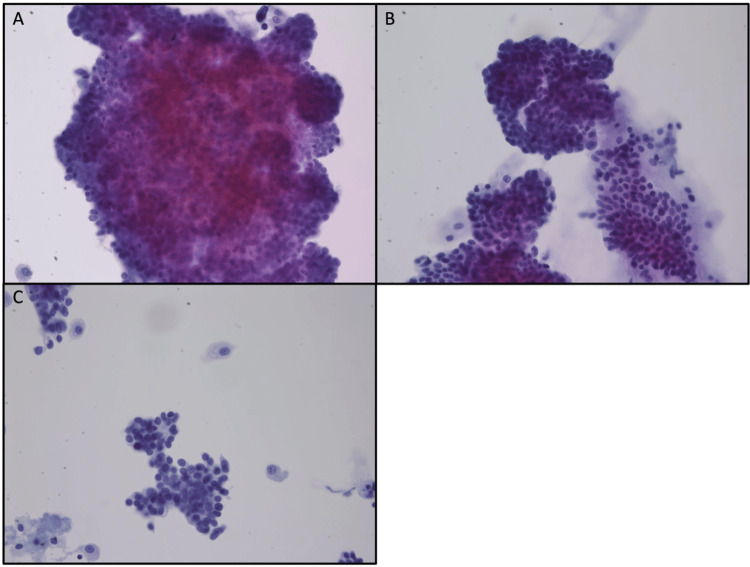
(A-C) Papanicolaou stained fine needle apirate smear showing clusters of cells with neuroendocrine features and minimal extracellular mucin (200× magnification).

**Figure 2:**
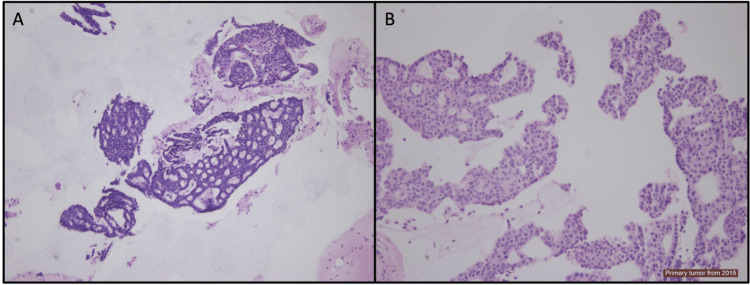
Hemotoxylin and eosin stained cell blocks from the current lesion (A) and previous (2015) lesion (B) showing similar cytologic features with solid aggregates including cribriform architecture of monomorphic cells with neuroendocrine features and eosinophilic cytoplasms. There is minimal intracellular and extracellular mucin identified in both specimens (100× magnification). Image (B) shows fading artifact of staining due to the age of the slide.

**Figure 3:**
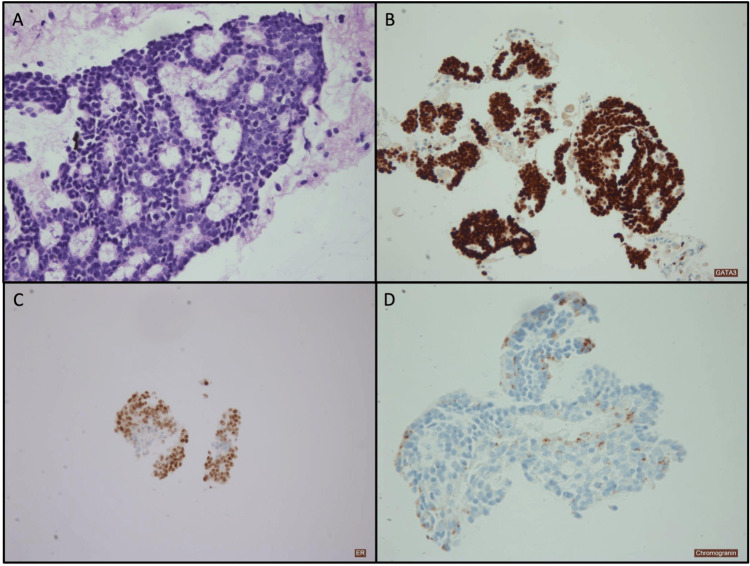
(A) Microscopy of cell block lesional tissue showing aggregates of monomorphic cells with neurendocrine features and cribriform architecture (hematoxylin and eosin; 200× magnification. Immunohistochemical stain demonstrating positive staining for (B) GATA3 (100× magnification); (C) ER (100× magnification); and (D) chromogranin (focal; 200× magnification).

A fine needle aspiration was performed on the lung lesion, and the cytology showed a cellular specimen with numerous large groups of hyperchromatic atypical cells with neuroendocrine features (Figure 1). Rare intranuclear inclusions were noted as suggestive of malignancy. A cell block was processed from the residual methanol-fixed specimen in this case. The cell block showed a cellular specimen with numerous large clusters of small cells with eosinophilic cytoplasm and inconspicuous nucleoli (Figure 2). No mitosis or necrosis was seen. The architecture showed a cribriform pattern in some areas. This morphology is similar to the patient’s prior tumour from 2015. Limited immunohistochemical stains demonstrate tumour cells staining positive for GATA3, ER, PR (focal), chromogranin (focal), and synaptophysin (focal) (Figure 3), with minimal mucin highlighted by the special stain mucicarmine. Lesional cells were negative for TTF1 and Napsin A.

The current case was compared to the previous EMPSGC, which showed identical cytologic features (Figure 2) and the same immunohistochemical staining pattern, so a limited panel was performed. Although immunohistochemical stains overlap with breast carcinoma, making a diagnosis challenging, the case was compared to the patient's prior intraductal papillary carcinoma and demonstrated varying histologic features. Although the patient had a history of metastatic prostate acinar adenocarcinoma, the immunohistochemical profile of the current limited panel was not in keeping with such a diagnosis. There was no evidence of any other lesions on imaging or physical exam to suggest a new primary, including breast or elsewhere. A final diagnosis of “metastatic endocrine mucin-producing sweat gland carcinoma of skin” was made, and after presentation at multi-disciplinary team (MDT) rounds and discussion with the patient, the plan was made for a thoracoscopic wedge resection of the lesion. This was performed on October 17, 2023. The results confirmed a metastatic EMPSGC.

## Discussion

Endocrine mucin-producing sweat gland carcinoma was previously believed to be a low-grade neuroendocrine neoplasm with an indolent clinical course and a low rate of recurrence (<10%) following excision [2,3,5,12]. There have now been seven reported cases since 2020 of metastases, including this case, with the majority metastasizing to the parotid gland and none metastasizing to the lungs [3,7,8]. It is important to differentiate this lesion from mucinous carcinoma and breast solid papillary carcinoma with neuroendocrine differentiation (SPCND). Mucinous carcinoma is differentiated due to its small clusters of cells with cribriform or tubular structures in large pools of extracellular mucin, whereas metastatic SPCND shows similar features and an immunohistochemical profile [2,3,13]. Although classically thought of as having more than 90% extracellular mucin, neuroendocrine and non-neuroendocrine mucinous carcinoma is now well known to exist in either pure form (>90%) or in a mixed pattern, which can lead to diagnostic confusion if the mucinous component is not properly sampled, as its presence, even in mixed form, should classify the lesion as a mucinous carcinoma [12,14,15]. This patient had a history of breast carcinoma, which allowed for the histological comparison of lesions for differentiation but would normally require clinical context and an appropriate workup.

EMPSGC is known to be a well-circumscribed, nodular, and multilobulated lesion with both solid and cystic areas and minimal intracellular and extracellular mucin [2,3]. The solid areas can also show rosette-like structures and focal cribriform architecture with apical snouts. It also has an inconspicuous and discontinuous myoepithelial cell layer surrounding lesional cells [2,3,7]. Cytological features of EMPSGC are not frequently described but show small to medium, round, monomorphic cells with neuroendocrine features such as finely granular or stippled nuclei and small or inconspicuous nucleoli in a background of minimal extracellular mucin [2,3,15]. Immunohistochemistry of EMPSGC shows lesional cells staining positive for CK7, CK8/18, AE1/AE3, CAM5.2, epithelial membrane antigen (EMA), E-cadherin, gross cystic disease fluid protein-15 (GCDFP-15), GATA3, WT1, oestrogen receptor, progesterone receptor, synaptophysin, chromogranin, neuron-specific enolase, and CD56, with occasional myoepithelial cells staining positive for CK5/6 and P63/P40 and a low Ki67 (<5%) [2-3,7,12]. Neuroendocrine markers can range from diffusely positive to focal or even absent in some specimens with limited tissue. There is minimal intracellular and extracellular mucin that can be highlighted with special stains such as mucicarmine or alcian blue [13]. There are no histologic or immunohistochemical features that are entirely specific to this entity and should be interpreted in the appropriate clinical context.

Although initially described in 1997, EMPSGC continues to be a rare entity, with only a low number of reported cases in the literature [5]. This histogenesis of EMPSGC is incompletely understood to date. The majority of reported cases have occurred within the last five to ten years, presumably due to their increased recognition [2,5,16]. As this is a rare tumour, there continues to be a lack of agreement as to whether this is a precursor to primary cutaneous mucinous carcinoma, on the spectrum of neuroendocrine mucinous carcinoma with minimal mucin, or a separate entity altogether. Thorough sampling is strongly encouraged to identify any variability in histomorphology and to reduce the risk of undersampling mutinous carcinoma. With the increasing recognition of this entity, true incidence rates and metastases will become more apparent. As reported cases have shown delayed presentations of metastases, the prognosis may be less indolent than previously believed, so prolonged follow-up may be necessary.

## Conclusions

This is the first reported case of EMPSGC with spread to the lung, adding to the literature on this rare neoplasm with emphasis on its recently described metastatic potential. As the number of reported cases of EMPSGC continues to grow, there will continue to be an increasing number of recognized cases of metastases. Continued recognition may also lead to more definitive criteria for the separation or combination of EMPSGC from or with primary mucinous carcinoma. Although currently rare, with the increased recognition of this entity, pathologists should keep this as part of their differential in mucinous and/or neuroendocrine neoplasms with thorough sampling, allowing for appropriate pathological and immunohistochemical workup. This case report contributes to the expanding knowledge of EMPSGC and underscores the importance of interdisciplinary collaboration between pathologists and clinicians in managing such complex and rare tumours.
